# Pioneering point-of-care obstetric ultrasound integration in midwifery education – the MEPOCUS study

**DOI:** 10.1186/s12909-024-06221-4

**Published:** 2024-10-24

**Authors:** Julia Groos, Adeline Walter, Ruben Plöger, Brigitte Strizek, Ulrich Gembruch, Agnes Wittek, Florian Recker

**Affiliations:** Department of Obstetrics and Prenatal Medicine, University Hospital Bonn, Venusberg Campus 1, 53127 Bonn, Germany

**Keywords:** Ultrasound, Training, Midwifery, Ultrasound curriculum, Point-of-care ultrasound, POCUS

## Abstract

**Background:**

Ultrasound technology is indispensable in perinatal care due to its non-invasive and painless nature, offering vital insights into foetal development and childbirth. With the academisation of midwifery in Germany, there is a growing necessity to incorporate ultrasound training into midwifery education. This paper discusses the development and implementation of an introductory obstetric ultrasound curriculum tailored for midwifery students, focusing on fundamental ultrasound techniques in obstetrics.

**Materials and methods:**

We used Kern’s six-step approach of curricular development comprising [1] problem identification and general needs assessment [2], needs assessment of the targeted learners [3], goals and objectives [4], educational strategies [5], implementation, and [6] evaluation and feedback. The individual components of the curriculum were meticulously designed based on comprehensive literature reviews, thorough consultations with experienced ultrasound experts and evaluated needs of participants prior to the course instruction.

**Results:**

Twenty-seven ultrasound-naive midwifery students participated in the newly developed obstetric ultrasound course. Structured as a modular and integrated framework, the course aimed to provide theoretical and practical instruction in basic obstetric ultrasound, with intrapartum sonography and focused assessment with abdominal sonography for trauma (FAST) as key supplementary specialisations. The results demonstrated a significant increase in the students’ overall knowledge and practical skills, as evidenced by the median post-course total score rising from 20 to 60 out of 75 (*p* < 0.001) in the objective structured clinical examination (OSCE) and from 9 to 19 out of 20 (*p* = 0.001) in the knowledge test. Additionally, students reported high satisfaction with the course and noted substantial personal benefits.

**Conclusion:**

The integration of basic obstetric ultrasound training within the midwifery curriculum is feasible and effective to teach fundamental knowledge and skills of obstetric ultrasound examinations to midwifery students. Expansion, standardisation and regulatory structures are critical components for a continued improvement and realistic integration into midwifery educational frameworks and thus the further development of the midwifery profession.

**Supplementary Information:**

The online version contains supplementary material available at 10.1186/s12909-024-06221-4.

## Background

The widespread utilisation of ultrasound as a diagnostic tool in pregnancy care has become increasingly prominent in recent years [1]. Its painlessness, radiation-free nature, and non-invasive character contribute to its high value, enabling the monitoring of foetal development and the well-being of both mother and child [2–4]. Especially, employing Point-of-Care Ultrasound (PoCUS) allows for flexible, precise, and rapid decision-making, hence its useful integration into daily practice within the delivery ward [1]. Here, the intrapartum usage of ultrasound enables diagnosing foetal head position and station and monitoring of head descent within the birth canal, thus providing assurance of favourable labour progress [5]. As a result, ultrasound technology assumes a pivotal role as an adjunctive tool to conventional diagnostic methods in both prenatal care and the delivery ward, thereby proving invaluable for midwives as well [4, 6].

Midwives, as key providers of prenatal care, shoulder significant responsibilities in providing care for pregnant women and their infants. Considering the value of midwives in obstetric care and the significance of ultrasound within this domain, it is prudent to universally integrate ultrasound training into midwifery education [7, 8]. From a global perspective, standardised ultrasound training for midwives has the potential to counteract the shortage of skilled ultrasound personnel, particularly prevalent in low- and middle-income countries (LMICs) [8]. This endeavour could help to better implement specific recommendations of the World Health Organization (WHO), by promoting access to early ultrasound examinations for pregnant women [9, 10]. In contrast to Germany, obstetric ultrasound has already become an integral component of midwifery practice in nations like Norway, where midwives obtain specialised qualifications as “midwife sonographers”, empowering them to perform ultrasound examinations independently [11]. However, with the implementation of the Midwifery Reform Act in 2020, the field of midwifery in Germany has experienced substantial changes, with a crucial step towards the academisation of midwifery. The ongoing academisation and elevation of qualification levels contribute to the development of midwifery work [12].

This trend positions ultrasound as an increasingly crucial element, with the potential to enhance the scope of midwifery practice, thereby facilitating the provision of comprehensive obstetric care. Although the foundation for the academic advancement of the midwifery profession is in place, consensus is still lacking on how to integrate and implement ultrasound training within midwifery education programmes. Therefore, our initiative seeks to initiate first steps towards the didactic incorporation of ultrasound training into midwifery education. The aim of the study was to establish a modular ultrasound course specifically designed for bachelor’s degree midwifery students and to assess the educational outcomes of this curriculum.

## Methods

The objective of this study was to develop an innovative ultrasound curriculum, utilising Kern’s six-step approach, for midwifery students of the bachelor’s degree programme in Midwifery Science at the Rhenish Friedrich-Wilhelms-University of Bonn, and to evaluate the course after its implementation [13]. The degree programme is structured as a dual bachelor’s programme with a standard duration of eight semesters. The ultrasound course is being implemented for the first time within the degree programme, aiming to equip third year midwifery students with both theoretical knowledge and practical skills through informative lectures and hands-on patient practice. Given the minimal inclusion of ultrasound training in the curriculum, most midwifery students possess little to no familiarity with ultrasound techniques, categorising them as novices in this area. Consequently, we utilised Kern’s six-step approach, which encompasses problem identification, needs assessment for targeted learners, setting goals and objectives, formulating educational strategies, implementation, and evaluation with feedback. Data acquisition and analysis were performed in compliance with protocols approved by the Ethical Committee of the University of Bonn (No. 179/23-EP). A declaration of consent was obtained from each study participant.

### Problem identification

In order to identify the most appropriate content for a comprehensive obstetric ultrasound curriculum for midwifery students, we undertook a detailed examination of several main sources, including the German Association of Midwives (Deutscher Hebammenverband, DHV), the European Midwives Association (EMA), the Global Midwives’ Hub, the International Confederation of Midwives (ICM) and the International Society for Ultrasound in Obstetrics and Gynecology (ISUOG). Additionally, we examined the published literature to research essential ultrasound competencies or algorithms deemed vital for midwives and to identify any potential ultrasound curricula already proposed for midwifery students.

### Needs assessment of targeted learners

Through a systematic literature review, we conducted an in-depth examination of international published literature to identify the specific needs of targeted learners within the relevant educational context [14]. Following this analysis, we discussed the identified content-specific, technical, and implementation-related needs with core group members, aligning them with the competencies proposed by DHV and ICM and tailoring them to our targeted learners. To ensure an adequate needs assessment, participating students were surveyed about their requirements before the course started, identifying a clear need that was also confirmed during the course’s implementation and taken into account accordingly. Additionally, a post-course survey was conducted to thoroughly assess the learners’ specific needs, enabling to effectively adapt and prioritise the content of potential subsequent courses.

### Goals and objectives

Identified content-specific, technical, and implementation-related needs were operationalised with regard to their technical, physiological, and pathological properties. As a result, goals and objectives were formulated through a synthesis of literature review, survey results, as well as the clinical expertise and previous evaluation of ultrasound curricula of panel members. We established a scientific Delphi process to develop the curriculum content and define learning objectives. This process involved clinical members possessing qualification levels I–III of the German Society for Ultrasound in Medicine (Deutsche Gesellschaft für Ultraschall in der Medizin, DEGUM) from the relevant specialty. These experts were integral to the Delphi methodology, which was conducted over two rounds. The process utilised multilevel, self-completed questionnaires based on a 9-point Likert scale, coupled with individual feedback sessions. The author collaborated with an additional ten members to facilitate this iterative method, ensuring a comprehensive and consensus-driven approach to curriculum development.

### Educational strategies

In order to address the defined learning goals and objectives, encompassing both theoretical knowledge and practical skills, various educational strategies were integrated in the curriculum. Within the selection process and with regard to the course content and structure, different educational methods were thoroughly analysed, compared and discussed. We used methods developed, used, and evaluated by the DEGUM for ultrasound training: lectures for the theoretical knowledge and supervised hands-on training for the practical scanning [15]. Furthermore, and in addition to traditional lectures, theoretical knowledge was delivered through a blended learning concept, allowing participants to access the teaching content digitally. The combined approach within the curriculum thus ensured comprehensive coverage of all areas and aspects.

### Implementation

The curriculum was delivered in two periods: from October 2023 to January 2024, and from April 2024 to July 2024, corresponding to the fifth and sixth semesters of the first academic year. Students were assigned to each semester in alphabetical order. As part of the curriculum development process, core group members participated in medical didactic training programmes to ensure they were well-prepared for the demands of implementing the curriculum. Board-certified experts in ultrasound and didactics, who had experience in ultrasound teaching, also contributed to the development and implementation of the curriculum [16]. Furthermore, the necessary infrastructure for the ultrasound curriculum was quickly established.

### Evaluation and feedback

To evaluate the attainment of predefined learning objectives and consequently gauge the success of the MEPOCUS curriculum, complementary methodologies were implemented at the start and completion of each course period, with an interval of three months between the respective evaluation points. These included an objective structured clinical examination (OSCE), administered in both semesters, as well as a theoretical single-choice test and a questionnaire, used in the second semester. All three evaluation methods were specifically developed for the purpose of this study. The knowledge test was designed in digital format and comprised a total of 20 questions covering key content of the seven modules of the course. For each question, students were required to choose the correct answer from four available options, with each correct answer earning one point, and a total possible score of 20 points. The OSCE consisted of three stations with clinical, case-based tasks solvable using course-acquired knowledge and skills, each worth 25 points, resulting in a total possible score of 75 points. Task sheets, assessment forms and instructions were tailored to defined objectives. Practical assessments in each room involved ultrasound examinations on a volunteer pregnant woman, evaluated by an obstetrics ultrasound specialist. Both the test and OSCE were applied in identical format before and after the course, with statistical analyses used to identify performance variances. Furthermore, a digital questionnaire was developed to collect students’ perspectives, self-reflection and feedback anonymously before the initial and after the final OSCE. The questionnaire included both Likert scale and open-ended questions. A 4-point Likert scale was used to evaluate students’ perspectives in the first part (1 = strongly disagree, 4 = strongly agree) and their self-assessment in the second part (1 = very unconfident, 4 = very confident). The questions were the same in both the pre- and post-course versions. As a third part, Likert styled questions were used in the pre-course questionnaire to gather midwives’ previous experiences with ultrasound (1 = strongly disagree, 4 = strongly agree) and in the post-course questionnaire to obtain course evaluations in combination with open-ended questions. All students and voluntary pregnant participants were comprehensively informed about the procedures prior to their participation, and informed consent was obtained from each individual.

### Statistical analysis

Statistical analysis was performed using Microsoft Excel software, version 2016 and SPSS (IBM SPSS Statistics for Windows, version 29.0.2.0). The results that were analysed, comprised the students’ performance on final OSCE, written test and questionnaire, compared to their initial assessments. Specifically, the students’ scores for each OSCE station and the total scores of pre- and post-course OSCE and test were reported as medians, mean values and standard deviations, with their 95% confidence interval. The normality of data distribution for the scores was evaluated using the Shapiro-Wilk test and histograms. Given the absence of normality in the data distribution, significant changes in pre- and post-course scores for all students with matched pre- and post-course scores were calculated using Wilcoxon signed-rank test. P values less than 0.05 were considered statistically significant.

## Results

An ultrasound curriculum specifically tailored for students of the inaugural Bachelor’s programme in Midwifery Sciences has been developed. The course’s emphasis on competency and quality is maintained by aligning with international and national guidelines, as well as engaging board-certified ultrasound experts and acclaimed didactic specialists. As a local initiative, our course represents an initial step in study-integrated ultrasound teaching for midwives.

### Problem identification

In absence of specific recommendations for ultrasound training tailored to midwives, several main sources increasingly adjust ultrasound proficiency to the essential competencies for basic midwifery practice. Accordingly, the International Confederation of Midwives (ICM) included the use of ultrasound to confirm pregnancy, estimate gestational age, and assess the well-being of both mother and child in its competency profile for midwives [17]. Similarly, the German Association of Midwives (Deutscher Hebammenverband, DHV) is dedicated to ensuring that graduates of the German midwifery degree programme attain proficiency in ultrasound application as an integral component of their academic training and has meticulously articulated this requirement within a carefully developed competency profile [18]. However, given the emergent nature of the topic in Germany, there is currently no standardised ultrasound curriculum integrated into the midwifery curriculum in Germany. Consequently, there exists a necessity for standardised ultrasound training programmes for midwifery students in Germany to achieve the competencies outlined by the ICM and DHV.

### Needs assessment of target learners

Following an extensive review of the international literature, alongside in-depth surveys, discussions, and meticulous adjustments, we systematically elucidated the needs of the participants in our ultrasound course. Accordingly, it was determined that midwifery students require an integrated educational approach that combines theoretical teaching with practical demonstration in group settings, as well as opportunities for independent application. Given their demanding schedules, it was also deemed essential that students have the ability to access course materials and engage with content independently, outside of the designated in-person events. Theoretical instruction was seen as a fundamental component, covering key topics such as ultrasound physics and technology, basic antepartum examinations like key biometric parameters, foetal presentation and foetal count, through to the evaluation of placental positioning and amniotic fluid volume [19]. Additionally, it was recognised that inclusion of essential intrapartum sonography elements and the focused assessment with sonography for trauma (FAST), according the FAST protocol, which includes the four standard exam views (the right upper quadrant, left upper quadrant, subcostal cardiac, and pelvis) presents a crucial element [20, 21]. In general, focusing on the fundamentals of ultrasound examination was deemed more effective than delving into detailed specifics. Hands-on training of theoretical content, guided by experienced ultrasound experts, was seen as a core component, necessitating a sufficient timeframe. Instruction and direct application on pregnant women were particularly valued for providing specific hands-on experience while fostering communicative skills and professional conduct. Furthermore, independent and autonomous ultrasound examinations in simulated scenarios were recognised as a vital component. The targeted needs assessment provided us with essential insights into prevailing pedagogical approaches, instructional content, and identified deficiencies that were taken into consideration during the implementation of our curriculum.

### Goals and objectives

**Fig. 1:**
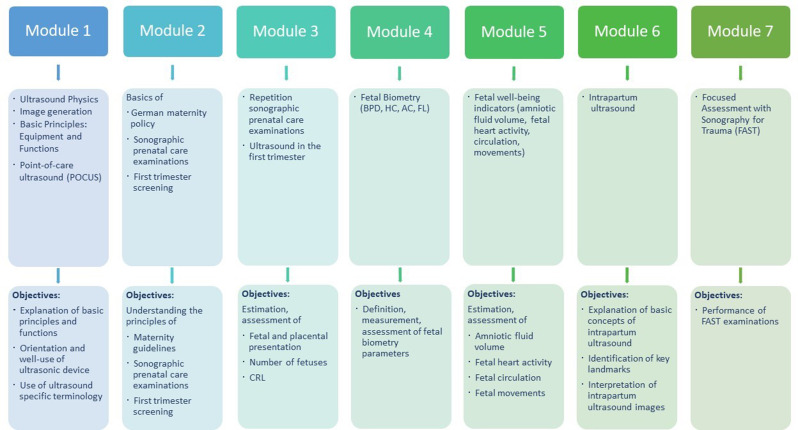
Modules and learning objectives of the obstetric ultrasound course. AC: abdominal circumference, BPD: biparietal diameter, CRL: crown rump length, FL: femur length, HC: head circumference

The needs assessment informed the structuring of the ultrasound curriculum into seven sequential modules. Each module was developed with specific knowledge-based and practical objectives that students are expected to achieve by the course’s conclusion. Figure 1 illustrates the learning objectives of each module, which cover fundamentals, as well as the specific topics of our course, the intrapartum sonography and FAST.

### Educational strategies

The seven modules of our ultrasound course encompassed both theoretical knowledge and practical competencies, necessitating the integration of diverse appropriate educational strategies (see Fig. 1). Theoretical knowledge is conveyed through a blended learning concept, consisting of classroom-based and online components. The initial two modules are exclusively provided online on the university’s learning platform, enabling asynchronous access that supports flexible study of fundamentals at their own pace and thus offers ideal preparation for application in class. The theoretical content of the remaining modules is delivered through traditional lectures at the beginning of each course day in-person, each conducted by a pair of team members who are both clinicians and experts in ultrasound. Following the course, the material from these modules remained accessible to students on the online platform. Additionally, they received an access code to a special e-learning system, where they could practise image recognition and interpretation at will and independently repeat the content learned in the course. In this practice-oriented course, the practical phase follows the theoretical lectures on each course day with a greater allocation of time and is conducted as supervised hands-on training sessions on voluntary pregnant women. During this phase of the course, students developed practical skills through a “See one, do one” approach, where ultrasound experts first demonstrated module-related content and then provided support and assistance to the students in carrying it out independently. The students were thus prepared to carry out independent, case-based ultrasound examinations in the final practical examination.

### Implementation

Our obstetric ultrasound curriculum for midwifery students was introduced by the local obstetrics department in 2023. The participating third-year students were divided into two cohorts. The first group, consisting of 14 students, attended the course in the winter semester of 2023/2024, while the second group, comprising 13 students, participated in the summer semester of 2024. The course scheduling was strategically planned to align with the students’ demanding timetables and the clinical commitments of the involved clinicians, ensuring the most suitable period for its execution. Key faculty members and essential stakeholders were engaged from the beginning of the project and the required infrastructure to sustain the curriculum was subsequently established. Each course day consisted of 60 min theoretical lectures and two hours practical training. Within the practical phases, students were divided into small groups of four to five persons, each supervised and supported by a tutor [16]. This division enabled each student to practise the module-related content and directly discuss the procedure and the images with the tutor. While practical exercises for module 7, covering the FAST domain, were conducted reciprocally among the students, pregnant women voluntarily participated in individual course sessions for all preceding modules, with their needs consistently taken into account.

### Evaluation and feedback

Within our obstetric ultrasound curriculum, a multifaceted assessment strategy was implemented, encompassing a pre-and post-course OSCE conducted over the entire project duration of two semesters, as well as a theoretical single-choice test and a questionnaire administered in the second semester. Each assessment approach was newly developed and administered both prior to and following the completion of the course, to effectively monitor the learning progress of knowledge, practical skills, and self-efficacy in conducting ultrasound examinations. The results of the different evaluation formats are detailed separately in the following sections.

### The pre-, post-course knowledge test

A single-choice knowledge test was conducted both before and after the course, using identical questions to ensure comparability. With a clear focus on evaluating the theoretical components of the course, two to three subject-specific questions were posed for each module. The questions consequently addressed the fundamentals of ultrasound knobology and handling, maternity guidelines, and foetal biometry, as well as foetal well-being monitoring, the utility of intrapartum ultrasound and the FAST examination. The comparative analysis of scores, with a maximum score of 20 points, shows that students’ total score levels were significantly lower in the pre-course knowledge test (Mdn = 9) than in the post-course knowledge test (Mdn = 19), z = -3.192, *p* = 0.001, *r* = -0.626 (see Table 1).

**Table 1:** Single-choice knowledge test results

Knowledge test	Median	Mean absolute	Standard deviation	Confidence interval
Pre	9	8.23	3.24	6.27–10.19
Post	19	16.54	3.95	14.15–18.93

### The pre-, post-course objective structured clinical examination

**Fig. 2:**
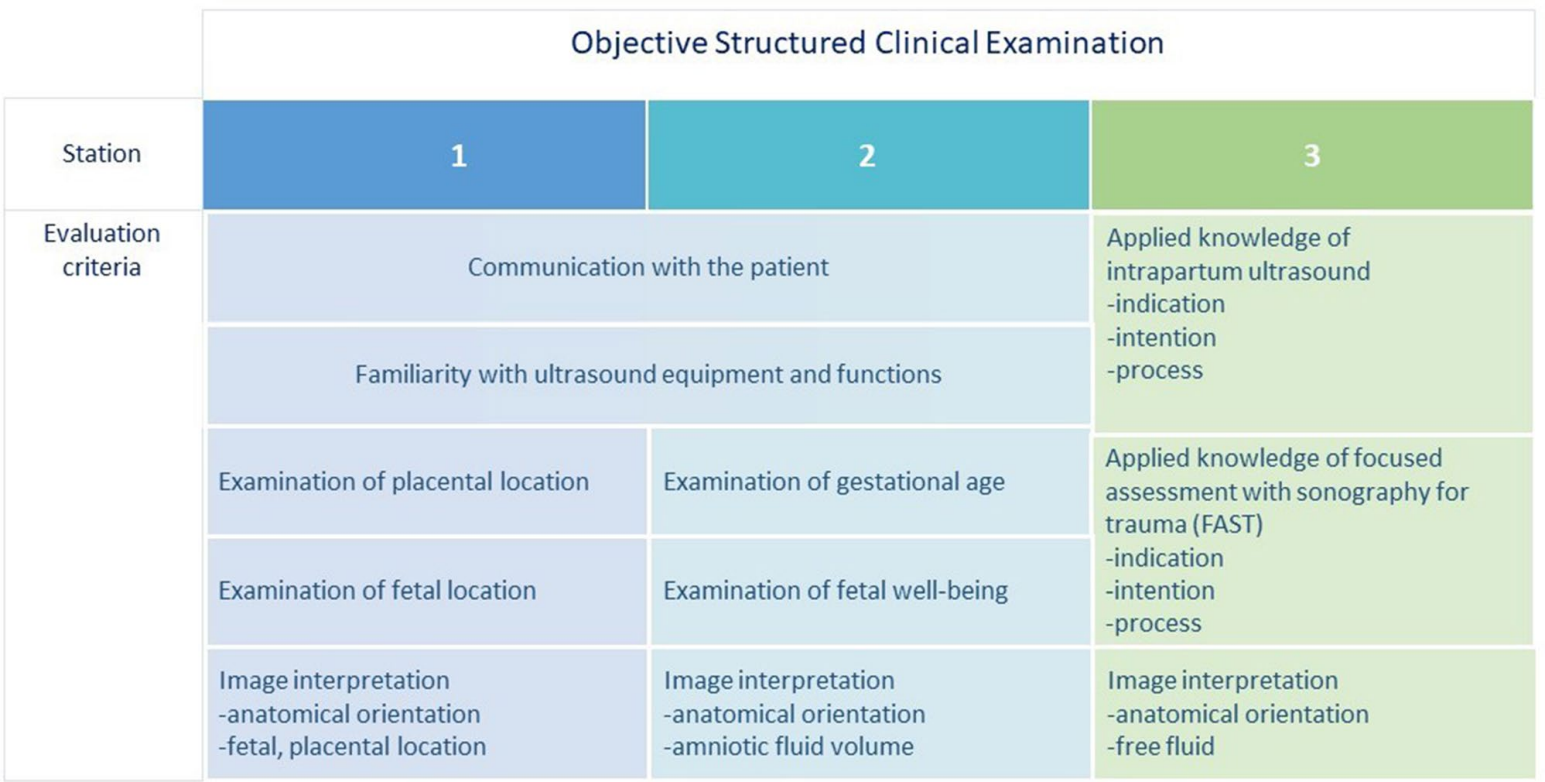
Evaluation criteria of Objective Structured Clinical Examination (OSCE)

The OSCE was successfully conducted both prior to and subsequent to the course using identical case-based tasks and assessment criteria to facilitate a direct performance comparison. Tasks were carried out with the involvement of three pregnant volunteers, each allocated to one station. Each station evaluated content from one of three course days in presence, with basic elements from modules 1 and 2 also included in every station’s evaluation (see Fig. 2). Due to absences in either the first or second OSCE, 23 of the 27 midwifery students could be included in the analysis. The comparative analysis of scores, with a maximum score of 75 points, shows that students’ total score levels were significantly lower on pre-course OSCE (Mdn = 20) than on post-course OSCE (Mdn = 60), z = -4.200, *p* < 0.001, *r* = -0.619. At the first station, akin to the subsequent two, participants underwent assessment on fundamental ultrasound operation techniques with the primary emphasis on the visualisation and localisation of the placenta and foetus. The results from this OSCE station show a significant rise in score from pre- (Mdn = 10) to post-course OSCE (Mdn = 23), z=-4.204, *p* < 0.001, *r* = -0,620. The second OSCE station focused on determining the gestational age using biometric parameters and indicators of foetal well-being, such as the amount of amniotic fluid in particular. The outcomes from this OSCE station also demonstrate a marked increase in score from pre- (Mdn = 6) to post-course OSCE (Mdn = 18), z = -4.202, *p* < 0.001, *r* = -0,620. At the third station, which focused on proficiency in intrapartum ultrasound and competencies in the FAST examination, results also indicate a significant increase in score from pre- (Mdn = 4) to post-course OSCE (Mdn = 23), z = -4.110, *p* < 0.001, *r* = -0,606. Correspondent differences of station-related pre- and post-course OSCE scores are illustrated in Table 2. The most significant improvement in scores between the pre- and post-course OSCE can be seen at station 3, indicating the greatest enhancement in knowledge and skills related to intrapartum sonography and FAST.

**Table 2:** Objective structured clinical examination (OSCE) results for all three stations

OSCE	Pre				Post			
Station	1	2	3	1–3 (total)	1	2	3	1–3 (total)
Median	10	6	4	20	23	18	23	60
Mean absolute	10.43	5.70	5.22	21.35	22.17	18.61	20.26	61.04
Standard deviation	1.73	2.38	3.63	5.25	1.72	4.50	6.04	9.32
Confidence interval	9.69–11.18	4.67– 6.73	3.65– 6.79	19.08– 23.62	21.43–22.92	16.45–20.77	17.65– 22.87	57.01– 65.07

### The pre-, post-course questionnaire

A digital questionnaire assessing participant perspectives, self-assessment, and satisfaction was administered to all 13 students (100%) of the second semester before and after the course. The pre-course questionnaire examined the students’ prior ultrasound experience and their perspectives on its application in midwifery, while the post-course version revisited these perspectives and included a course evaluation. Identical self-assessment questions in both questionnaires highlighted potential performance improvements, evaluating perceived confidence in using ultrasound technology and performing specific examinations. These covered proficiency in handling the ultrasound probe and knobology, visualising the foetus, placenta and uterine artery, measuring biometric parameters and amniotic fluid volume, conducting the FAST examination. The questionnaire results indicate that the majority (*n* = 12, 92.3%) had no prior experience with ultrasound. Nevertheless, all students initially either agreed (*n* = 6, 46.2%) or strongly agreed (*n* = 7, 53.8%) that they were interested in integrating ultrasound in their clinical practice. Pre-course self-evaluation shows all students had low or very low confidence in all application areas, particularly they reported very low confidence in knobology (*n* = 13, 100%), measuring foetal head and abdominal circumference (*n* = 12, 92.3%), measuring femur length (*n* = 11, 84.6%), and conducting the FAST examination (*n* = 13, 100%). Post-course results indicate an overall increase in confidence. Especially, students felt confident or very confident in handling the ultrasound transducer (*n* = 9, 69.2%), visualising the foetus (*n* = 11, 84.6%), measuring amniotic fluid volume (*n* = 9, 69.2%), and conducting the FAST examination (*n* = 8, 61.6%). However, few students still felt very unconfident in knobology (*n* = 3, 23.1%) and measuring femur length (*n* = 3, 23.1%). The course evaluations indicate that participants found the course helpful in improving their ultrasound skills and confidence, and that they found the focus of the training on the basics appropriate. Students found comprehensive device operation, including knobology, the orientation in the image and measuring femur length particularly challenging. Many emphasised the importance of hands-on training and expressed a desire for more sessions to better apply and consolidate their learning.

## Discussion

As a response to the evolving demands of the midwifery profession, this study represents the inaugural effort to develop and implement a comprehensive obstetric ultrasound curriculum within a midwifery degree programme in Germany. Our study shows that incorporating ultrasound instruction within midwifery education is practicable and significantly improves students’ theoretical knowledge and practical skills. Successes were assessed using an OSCE, a knowledge-based single-choice test, and a self-assessment questionnaire, all administered both before and after the course.

Several other studies have been published on training courses for midwives in the field of obstetric ultrasound, demonstrating comparable successes [9, 22, 23]. For instance, Bentley et al. reported on a one-week ultrasound curriculum for midwives in Liberia, which resulted in a notable increase in the midwives’ knowledge and practical skills, as well as in self-rated comfort [24]. In the spectrum of existing comparable initiatives, our research significantly contributes to the field, as most existing studies, identified in our systematic literature review, have been conducted in low- and middle-income countries, where intentions, frameworks, and resource availability differ significantly from those in industrialised nations and thus contribute to significant challenges in course instruction [14]. Our ultrasound course benefited from advanced infrastructure and abundant resources, allowing for a different approach with broad implications for midwifery education. Our project targets midwives in training and integrates seamlessly into their curriculum, unlike most studies that include practising midwives, often leading to conflicts with their professional responsibilities [9, 25]. We are therefore following the recommendation of Hall et al., which suggests to establish dedicated timeslots for attending practical sessions to ensure they do not overlap with other commitments [22].

To ensure the success of ultrasound courses, the implementation necessitates several considerations, with a key one being a needs assessment to identify participants’ requirements, which in turn shapes the learning objectives, content, and course structure [13]. Our needs assessment led to a blended learning approach with fundamental theoretical and practical content, supplemented by online materials and autonomous ultrasound examinations in simulated scenarios [14]. Each of these aspects, particularly the combination of didactic and hands-on elements, has proven effective in previous projects [23, 26, 27]. This blend of learning methods facilitated a modular structure of the course, leading to effective content organisation and the inclusion of both online and face-to-face course days. Unlike Shaw-Battista et al., we used online modules only to introduce basic knowledge, reserving face-to-face sessions for advanced and practical content [28]. Our emphasis on foundational instruction, encompassing ultrasound machine operation and fundamental obstetric examination techniques, has been well-regarded in the literature and highly valued by our participants [9, 29]. Additionally, we uniquely included instruction in FAST and intrapartum sonography, equipping participants with crucial skills for obstetric scenarios [30, 31]. In this context, transabdominal intrapartum sonography is particularly notable for its relative ease of learning compared to conventional clinical methods for determining foetal position during labour [32]. As a highly valued aspect noted by Shaw-Battista et al., the practical instruction included supervised hands-on practice with experienced ultrasound professionals [28]. Finally, the combination of test, practical examination, and questionnaire, an approach that had already been successfully applied in further researches, enabled a comprehensive evaluation of the students’ learning progress [9, 24].

Despite the successful implementation, our study has several limitations, particularly the constrained timeframe due to integrating teaching units into the extensive bachelor’s programme curriculum, which limited topic coverage, repetition, and time for developing practical skills. Similar to findings by Shah et al., students highlighted this deficit, suggesting extended or more frequent sessions would have been beneficial [23, 33]. Evaluations were conducted three months apart, covering a large amount of material in a short period, making it challenging to achieve high proficiency in all areas. Skills with high redundancy, such as device operation, image orientation, and foetal location, are likely to be retained longer than less frequently covered but essential topics like biometric measurements. While our study demonstrates comprehensive knowledge gains, it remains unclear how well these skills will be retained and applied long term, particularly outside the structured learning environment, highlighting the need for long-term observation studies. Especially, first trimester ultrasound, as a comprehensive core procedure, may require advanced expertise and extended focused training to ensure thorough and safe coverage. Although students will have gained an insight into first trimester ultrasound within two modules, it seems unlikely that they have achieved the necessary competence to perform these examinations independently. Additionally, learning outcomes during the practical exercises varied due to the randomised assignment of groups and pregnant participants, limiting exposure to diverse clinical scenarios, which may affect recognition in actual patients post-course. Staff shortages also led to different raters in the pre- and post-course OSCE, potentially impact interrater reliability.

Although midwifery education and associated ultrasound training at German universities are still in their infancy, policy interventions are needed to expand academisation and enhance access to advanced educational opportunities [14]. In addition to firmly integrating ultrasound training into the curricula with sufficient time to cover foundational topics, early establishment of alignments and basic standards is essential for ensuring consistent competency, improving educational methods, and developing the midwifery practice [12, 14]. Furthermore, regulatory measures, including financing teaching costs, setting guidelines for role allocations, and funding ultrasound examinations by midwives, are crucial [14].

Ultrasound proficiency is gaining increasing significance for midwifery students from both registration and workplace perspectives. In many regions, regulatory bodies and professional standards for midwives increasingly recognise ultrasound as part of a midwife’s scope of practice. While not universally required as a core competency, there is a growing expectation for midwives to possess foundational knowledge in ultrasound, particularly in placental location, foetal positioning, and amniotic fluid assessment. From a registration perspective, ultrasound competency can elevate professional qualifications, aligning with the evolving needs of healthcare systems, and as the use of ultrasound technology in routine antenatal care increases, midwifery students skilled in ultrasound techniques may experience better job prospects. Employers often prefer candidates who offer a broader range of services, such as ultrasound, allowing for more comprehensive diagnostics and fewer referrals. In the workplace, ultrasound allows midwives to provide more comprehensive antenatal and emergency care, enabling quicker clinical decisions, earlier interventions and more personalised birth planning. However, with transitions of students into clinical practice, it is essential to conduct long-term observations to assess the practical application of ultrasound skills, how these responsibilities are coordinated with other professional groups such as physicians, and how ultrasound education for midwives can be adapted accordingly.

## Conclusion

Our study represents the inaugural initiative to introduce a comprehensive obstetric ultrasound curriculum into a midwifery degree programme in Germany, demonstrating its viability and effectiveness in training midwifery students. Statistical analyses reveal significant enhancements in the students’ knowledge and skills across all areas of the course, suggesting that the curriculum serves as a valuable teaching approach and provides well-tailored educational strategies that meet the specific needs of the students. Establishing early standards and developing comprehensive financial and regulatory frameworks are crucial to further improve and realise the firm integration of ultrasound teaching in midwifery education. Finally, the incorporation of ultrasound training may constitute an important component in the ongoing process of professionalising and academising the field of midwifery.

## Electronic supplementary material

Below is the link to the electronic supplementary material.

Supplementary Material 1

Supplementary Material 2

Supplementary Material 3

## Data Availability

Data available on request from the authors.
