# Supplementary material for: Pioneering point-of-care obstetric ultrasound integration in midwifery education – the MEPOCUS study
Source: BMC Med Educ. 2024 Oct 24;24:1209. doi: 10.1186/s12909-024-06221-4 (PMC11515421; doi:10.1186/s12909-024-06221-4)
Supplement: Supplementary file 1 — Supplementary Material 1 [file 12909_2024_6221_MOESM1_ESM.pdf]

Pre-course questionnaire

**Your opinion**	1<br>strongly disagree	2<br>disagree	3<br>agree	4<br>strongly agree
I consider obstetric ultrasound diagnostics to be a highly valuable and effective modality.				
I would integrate sonography into my routine clinical practice if the necessary resources were available.				
**Your subjective competency assessment**	1<br>very unconfident	2<br>unconfident	3<br>confident	4<br>very confident
How would you rate your overall confidence in the application of ultrasound?				
How confident are you in utilizing ultrasound within the following areas?				
Handling the ultrasound transducer				
Operating the ultrasound machine, including the knobology				
Visualizing the fetus				
Determining fetal position				
Determining placental location				
Measuring fetal head and abdominal circumference				
Measuring femur length				
Locating the uterine artery				
Assessing amniotic fluid volume				
Performing a FAST (Focused Assessment with Sonography for Trauma) examination				
**Your experiences**	1<br>strongly disagree	2<br>disagree	3<br>agree	4<br>strongly agree
I have experience in performing ultrasound examinations.				
I have already participated in training programs in the field of ultrasound.				
I am interested in enhancing my skills in ultrasound diagnostics.				
