# Supplementary material for: Pioneering point-of-care obstetric ultrasound integration in midwifery education – the MEPOCUS study
Source: BMC Med Educ. 2024 Oct 24;24:1209. doi: 10.1186/s12909-024-06221-4 (PMC11515421; doi:10.1186/s12909-024-06221-4)
Supplement: Supplementary file 2 — Supplementary Material 2 [file 12909_2024_6221_MOESM2_ESM.pdf]

<u>The pre- and post-course test</u>

1. In a longitudinal ultrasound scan, in which direction should the marker on the transducer be oriented?

☐ Towards the patient's feet (caudal)

☐ Towards the patient's head (cranial)

☐ To the patient's right side

☐ To the patient's left side

2. Which type of transducer is most commonly used for abdominal sonography?

☐ Linear transducer

☐ Sector transducer

☐ Convex transducer

☐ Flexor transducer

3. How can the brightness of the ultrasound image be adjusted?

☐ By modifying the gain setting

☐ By adjusting the ultrasound frequency

☐ By changing the distance between the transducer and the patient

☐ By altering the room lighting

4. According to the maternity guidelines, when is the first of the three standard ultrasound examinations during pregnancy typically performed?

☐ Between 4+0 and 7+6 weeks of gestation

☐ Between 10+0 and 13+6 weeks of gestation

☐ Between 6+0 and 9+6 weeks of gestation

☐ Between 8+0 and 11+6 weeks of gestation

5. According to the maternity guidelines, which of the following is not a standard parameter for fetal biometry during the three routine ultrasound examinations?

☐ Biparietal diameter

☐ Abdominal circumference

☐ Crown-rump length

☐ Leg circumference

6. Which biometric parameter is typically the first to be measured during early pregnancy?

- Biparietal diameter
- Femur length
- Crown-rump length
- Abdominal circumference

7. During an ultrasound examination of a pregnant woman, you observe that the fetus's back is visualized on the left side of the image in the transverse plane. What is the position of the fetus?

- First position: back to the left, anterior fontanelle to the right
- First position: back to the right, anterior fontanelle to the left
- Second position: back to the right, anterior fontanelle to the left
- Second position: back to the left, anterior fontanelle to the right

8. Which structure is typically measured during the first-trimester ultrasound screening?

- Diameter of the fetal heart
- Nuchal translucency
- Volume of the fetal brain
- Width of the fetal nose

9. What is the biparietal diameter?

- The maximum transverse diameter of the fetal pelvis
- The maximum transverse diameter of the fetal abdomen
- The maximum transverse diameter of the fetal head
- The maximum transverse diameter of the fetal thorax

10. How are measured fetal biometric parameters assessed as normal or pathological?

- By comparing them with the parameters from the previous ultrasound of the same fetus
- By the subjective evaluation of the ultrasound technician
- By comparing them with percentiles based on population data
- By comparing them with the parents' measurements at the same gestational age

11. Which of the following abnormal fetal measurements is most indicative of intrauterine growth restriction (IUGR)?

- [ ] A reduced head circumference
- [ ] Insufficient blood flow to the fetal head
- [ ] A reduced abdominal circumference
- [ ] An excessively high heart rate

12. What is the Amniotic Fluid Index (AFI)?

- [ ] The highest measured density value of the amniotic fluid in any of the four quadrants of the pregnant abdomen
- [ ] The sum of the respective measured density values of the amniotic fluid in all of four quadrants of the pregnant abdomen
- [ ] The deepest measured amniotic fluid pocket in any of the four quadrants of the pregnant abdomen
- [ ] The sum of the deepest measured amniotic fluid pockets in all four quadrants of the pregnant abdomen

13. From which gestational week is fetal viability typically confirmed by detecting heart activity via ultrasound?

- [ ] From the 2nd week of pregnancy
- [ ] From the 4th week of pregnancy
- [ ] From the 6th week of pregnancy
- [ ] From the 9th week of pregnancy

14. What is the most effective method for visualizing the maternal uterine artery using ultrasound?

- [ ] Position the probe in the groin area parallel to the longitudinal axis and tilt slightly medially
- [ ] Position the probe in the groin area parallel to the longitudinal axis and tilt slightly laterally
- [ ] Position the probe in the groin area parallel to the transverse axis and tilt slightly medially
- [ ] Position the probe in the groin area parallel to the transverse axis and tilt slightly laterally

15. What parameter is measured to assess the progress of labor during the intrapartum period?

- [ ] Degree of Delivery (DoD)
- [ ] Curve of Progression (CoP)
- [ ] Index of Delivery (IoD)

- [ ] Angle of Descent (AoD)

16. What does the abbreviation ITU stand for?

- [ ] Intrapartum Translabial Ultrasound
- [ ] Intrauterine Tissue Ultrasound
- [ ] Intrapartum Transabdominal Ultrasound
- [ ] Interstitial Transuterine Ultrasound

17. What can be effectively measured using ITU?

- [ ] The frequency of uterine contractions during labor
- [ ] The height and direction of the fetal head in relation to the maternal pelvis
- [ ] The rotation of the fetal shoulders during birth
- [ ] The elasticity of the cervix during childbirth

18. What is primary objective during a sonographic FAST examination?

- [ ] Detection of inflammations of internal organs
- [ ] Identification of bone fractures
- [ ] Assessment of blood circulation disorders in large arteries
- [ ] Detection of free fluid

19. Which of the following views is not part of the standard FAST protocol?

- [ ] Right flank view
- [ ] Left flank view
- [ ] Suprapubic transverse view
- [ ] Infrapubic longitudinal view

20. What is referred to as Morison's pouch?

- [ ] The space between the liver and kidney
- [ ] The space between the spleen and kidney
- [ ] The space between the uterus and bladder
- [ ] The space between the uterus and rectum
