# Supplementary material for: Pioneering point-of-care obstetric ultrasound integration in midwifery education – the MEPOCUS study
Source: BMC Med Educ. 2024 Oct 24;24:1209. doi: 10.1186/s12909-024-06221-4 (PMC11515421; doi:10.1186/s12909-024-06221-4)
Supplement: Supplementary file 3 — Supplementary Material 3 [file 12909_2024_6221_MOESM3_ESM.pdf]

Post-course questionnaire

Your opinion	1<br>strongly disagree	2<br>disagree	3<br>agree	4<br>strongly agree
I consider obstetric ultrasound diagnostics to be a highly valuable and effective modality.				
I would integrate sonography into my routine clinical practice if the necessary resources were available.				
**Your subjective competency assessment**	1<br>very unconfident	2<br>unconfident	3<br>confident	4<br>very confident
How would you rate your overall confidence in the application of ultrasound?				
How confident are you in utilizing ultrasound within the following areas?				
Handling the ultrasound transducer				
Operating the ultrasound machine, including the knobology				
Visualizing the fetus				
Determining fetal position				
Determining placental location				
Measuring fetal head and abdominal circumference				
Measuring femur length				
Locating the uterine artery				
Assessing amniotic fluid volume				
Performing a FAST (Focused Assessment with Sonography for Trauma) examination				
**Your course evaluation**	1<br>strongly disagree	2<br>disagree	3<br>agree	4<br>strongly agree
Overall, I found the ultrasound course to be satisfactory.				
I believe that the course has enhanced my knowledge in obstetric ultrasound.				
I feel that the course has increased my confidence in operating the ultrasound machine.				

I would have preferred more course sessions.				
I would recommend the ultrasound course to my peers/colleagues.				
The following aspects of the course were the most helpful for me:				
The following aspects of the course were the most challenging for me:				
I would like to see the following topics or techniques covered in future courses:				
In my opinion, the course could be improved in the following areas:				
